# Control of Human T-Cell Leukemia Virus Type 1 (HTLV-1) Infection by Eliminating Envelope Protein-Positive Cells with Recombinant Vesicular Stomatitis Viruses Encoding HTLV-1 Primary Receptor

**DOI:** 10.1128/JVI.01885-17

**Published:** 2018-01-30

**Authors:** Kenta Tezuka, Kazu Okuma, Madoka Kuramitsu, Sahoko Matsuoka, Reiko Tanaka, Yuetsu Tanaka, Isao Hamaguchi

**Affiliations:** a Department of Safety Research on Blood and Biological Products, National Institute of Infectious Diseases, Tokyo, Japan; b Department of Immunology, Graduate School of Medicine, University of the Ryukyus, Okinawa, Japan; Ulm University Medical Center

**Keywords:** HTLV-1 Env-expressing cells, HTLV-1 carrier, HTLV-1 infection, HTLV-1 receptor molecule, humanized mouse, recombinant VSV, targeting virotherapy

## Abstract

Human T-cell leukemia virus type 1 (HTLV-1) infection causes adult T-cell leukemia (ATL), which is frequently resistant to currently available therapies and has a very poor prognosis. To prevent the development of ATL among carriers, it is important to control HTLV-1-infected cells in infected individuals. Therefore, the establishment of novel therapies with drugs specifically targeting infected cells is urgently required. This study aimed to develop a potential therapy by generating recombinant vesicular stomatitis viruses (rVSVs) that lack an envelope glycoprotein G and instead encode an HTLV-1 receptor with human glucose transporter 1 (GLUT1), neuropilin 1 (NRP1), or heparan sulfate proteoglycans (HSPGs), including syndecan 1 (SDC1), designated VSVΔG-GL, VSVΔG-NP, or VSVΔG-SD, respectively. In an attempt to enhance the infectivity of rVSV against HTLV-1-infected cells, we also constructed rVSVs with a combination of two or three receptor genes, designated VSVΔG-GLN and VSVΔG-GLNS, respectively. The present study demonstrates VSVΔG-GL, VSVΔG-NP, VSVΔG-GLN, and VSVΔG-GLNS have tropism for HTLV-1 envelope (Env)-expressing cells. Notably, the inoculation of VSVΔG-GL or VSVΔG-NP significantly eliminated HTLV-1-infected cells under the culture conditions. Furthermore, in an HTLV-1-infected humanized mouse model, VSVΔG-NP was capable of efficiently preventing HTLV-1-induced leukocytosis in the periphery and eliminating HTLV-1-infected Env-expressing cells in the lymphoid tissues. In summary, an rVSV engineered to express HTLV-1 primary receptor, especially human NRP1, may represent a drug candidate that has potential for the development of unique virotherapy against HTLV-1 *de novo* infection.

**IMPORTANCE** Although several anti-ATL therapies are currently available, ATL is still frequently resistant to therapeutic approaches, and its prognosis remains poor. Control of HTLV-1 *de novo* infection or expansion of HTLV-1-infected cells in the carrier holds considerable promise for the prevention of ATL development. In this study, we developed rVSVs that specifically target and kill HTLV-1 Env-expressing cells (not ATL cells, which generally do not express Env *in vivo*) through replacement of the G gene with HTLV-1 receptor gene(s) in the VSV genome. Notably, an rVSV engineered to express human NRP1 controlled the number of HTLV-1-infected Env-expressing cells *in vitro* and *in vivo*, suggesting the present approach may be a promising candidate for novel anti-HTLV-1 virotherapy in HTLV-1 carriers, including as a prophylactic treatment against the development of ATL.

## INTRODUCTION

Human T-cell leukemia virus type 1 (HTLV-1) belongs to the genus Deltaretrovirus and the family Retroviridae and is a spherical virus with a nonsegmented, positive-stranded RNA genome (1, 2). HTLV-1 infection is endemic in southern Japan, the southern United States, central Australia, the Caribbean, Jamaica, South America, and equatorial Africa (3). HTLV-1 causes related diseases, such as adult T-cell leukemia (ATL), HTLV-1-associated myelopathy/tropical spastic paraparesis (HAM/TSP), and HTLV-1 uveitis in humans after a long latent infection, although most HTLV-1-infected individuals (carriers) are asymptomatic (4). HTLV-1 transmits efficiently through cell-cell contact, but not by a cell-free mechanism, and infects humans via three main routes: vertical (from mother to infant, mostly by breast-feeding), horizontal (sexual), and parenteral transmission (transfusion) (5–7). A previous national survey in Japan reported approximately 1,080,000 asymptomatic carriers (8), indicating that the total number of carriers has gradually decreased and that measures against mother-to-infant transmission are effective. However, a recent report predicted that more than 4,000 new infections have occurred in Japan, suggesting that some measure against horizontal infection is needed (9). Also, although several anti-ATL therapies are currently available, including chemotherapy, allogeneic bone marrow transplantation (10), and anti-CCR4 antibody (11), ATL is frequently resistant to these treatments, and its prognosis remains poor (12). The Joint Study on Predisposing Factors of ATL Development (JSPFAD) reported that carriers with a proviral load (PVL) exceeding 4% (4 copies/100 cells) may be a high-risk group in whom ATL develops (13). Thus, to follow up the carriers, control of HTLV-1 infection/HTLV-1-infected cells is important and urgently required as an active intervention for HTLV-1-infected individuals.

After entering human host cells, HTLV-1 exists as a provirus (proviral DNA) integrated into the human genome, and *gag*, *pro*, *pol*, *env*, and *pX* are transcribed from the HTLV-1 genome (14). The *env* gene codes for envelope glycoproteins (Env, gp46, and gp21) that are responsible for the specific binding of HTLV-1 to cellular receptor(s) and catalyzing virus-cell membrane fusion in a pH-nondependent manner, leading to viral entry into host cells (15). Because HTLV-1 Env is expressed from the provirus on the surface of infected cells, the formation of giant multinucleated cells termed syncytium is induced at least *in vitro* by cell-cell fusion following interactions between Env on infected cells and receptor(s) on neighboring noninfected cells (16). This induction appears to depend on cell types and mediates cell death in formed syncytia. HTLV-1 primarily infects and immortalizes human CD4 T cells, but *in vitro*, it infects many cell types derived from various species (17). This wide host range indicates that a primary receptor for HTLV-1 entry should be a molecule ubiquitously expressed on the cell surface, and indeed glucose transporter 1 (GLUT1), neuropilin 1 (NRP1), and heparan sulfate proteoglycans (HSPGs), including syndecan 1 (SDC1), were identified as HTLV-1 surface receptors (18–20).

Vesicular stomatitis virus (VSV) belongs to the genus Vesiculovirus and the family Rhabdoviridae and is composed of the Indiana, New Jersey, and Alagoas serotypes (21, 22). The main host of VSV is livestock, such as horses, cattle, and swine (23). VSV is a bullet-shaped virus with a nonsegmented, negative-stranded RNA genome that encodes five structural proteins (N, P, M, G, and L) (24). VSV G is the only envelope glycoprotein, which attaches VSV to a cell surface receptor of host cells and catalyzes pH-dependent viral entry into the cells (25). VSV infects various cell types *in vitro*, replicates, produces a number of progeny virions, and rapidly induces cytolysis in infected cells (26).

It is well known that VSV can be recovered from plasmid DNA copies. The VSV genome can also be engineered such that foreign genes are inserted and expressed in recovered viruses (27). To establish VSV-based antiviral strategies (platform) called “virotherapy,” we have engineered and developed recombinant VSVs (rVSVs) aimed at targeting and eliminating human immunodeficiency virus type 1 (HIV-1)-infected or simian immunodeficiency virus (SIV)-infected cells (28–30). In the previous studies, we have successfully generated rVSVs in which HIV-1/SIV receptors were substituted with VSV G. These foreign molecules were expressed by the VSV genome and incorporated on the envelopes of produced viral particles instead of VSV G. Because HIV-1/SIV-infected cells express viral envelope glycoproteins on the cell surface, the binding of HIV-1/SIV receptors on rVSVs with HIV-1/SIV envelope glycoproteins on the target infected cells triggered virus-cell membrane fusion catalyzed by the envelope glycoproteins, leading to specific entry (superinfection) of the rVSVs into the target cells. Consequently, these rVSV replicates exerted a strong cytolytic effect and eliminated HIV-1/SIV-infected cells selectively, which finally controlled HIV-1/SIV infection *in vitro* and/or *in vivo* (28–30).

This study established VSV-based anti-HTLV-1 virotherapy by engineering and generating novel rVSVs lacking G and instead encoding/expressing HTLV-1 receptor(s). These rVSVs were expected to target and attack HTLV-1-infected Env-expressing cells (not ATL cells, which generally do not express Env *in vivo*) via the interplay between the receptor(s) incorporated on rVSVs and the Env expressed on the surface of infected target cells. We tested whether the novel rVSVs entered, replicated, and eliminated HTLV-1-infected Env-expressing cells specifically, leading to the control of HTLV-1 *de novo* infection *in vitro* and *in vivo*. We then evaluated the therapeutic potential of these rVSVs.

## RESULTS

### Construction and generation of rVSVs expressing HTLV-1 receptor molecule(s).

In the present study, we generated VSV recombinants expressing HTLV-1 receptor(s) to target HTLV-1-infected cells that express Env protein. Numerous studies have suggested that GLUT1, NRP-1, and HSPGs are receptor candidates for HTLV-1 infection (18–20). Recently, an important study revealed that SDC1, a member of the HSPG core protein, played a significant role in cellular susceptibility to HTLV-1 (31). Based on these findings, we aimed to express the GLUT1, NRP1, and/or SDC1 protein on the envelope of rVSVs to target and superinfect HTLV-1-infected Env-expressing cells through the interaction with receptor molecules and HTLV-1 Env expressed on the cell surface of target cells.

**FIG 1:**
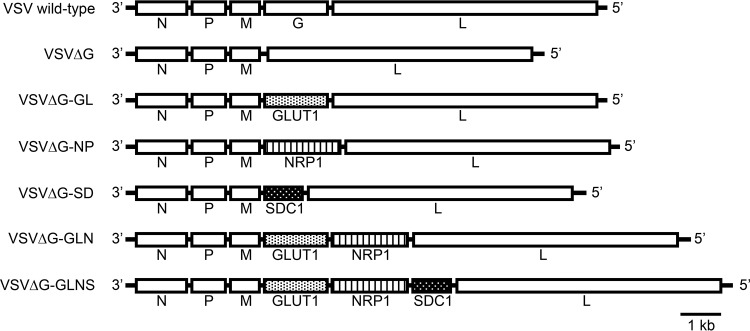
Schematic structure of rVSVs expressing HTLV-1 receptor molecule(s). The gene orders in the rVSV constructs used in this study and wild-type construct are illustrated. The VSVΔG construct lacks the VSV G gene, which encodes a VSV envelope glycoprotein. The deleted G gene was replaced with human GLUT1, NRP1, or SDC1 genes, yielding the VSVΔG-GL, VSVΔG-NP, or VSVΔG-SD constructs, respectively. In addition, a human NRP1 gene was inserted downstream of the GLUT1 gene in VSVΔG-GL, yielding VSVΔG-GLN. The human SDC1 gene was inserted downstream of the NRP1 gene in VSVΔG-GLN, yielding VSVΔG-GLNS.

To address this issue, we initially constructed an rVSV expressing the human GLUT1, NRP1, or SDC1 gene. The G gene was deleted from the VSV wild-type (Indiana strain) plasmid construct (pVSV-XN2), and each HTLV-1 receptor gene was inserted between the M and L genes as a substitute for the deleted G gene. The infectious viruses recovered from these constructs were designated VSVΔG-GL, VSVΔG-NP, or VSVΔG-SD, respectively (Fig. 1). Next, the gene encoding NRP1 was inserted downstream of the GLUT1 gene in VSVΔG-GL in an attempt to increase the functional interaction of receptor molecules with the Env protein. The infectious virus recovered from the construct was designated VSVΔG-GLN (Fig. 1). Moreover, the gene encoding SDC1 was inserted downstream of the NRP1 gene in VSVΔG-GLN, and the recovered virus was designated VSVΔG-GLNS (Fig. 1). Because the G gene was deleted from the viral genome, the constructed viruses were initially produced in the presence of complementing G protein with BHK-21 cells expressing G protein (32).

### Cell surface expression of HTLV-1 receptor molecule(s) encoded in the rVSV genome.

**FIG 2:**
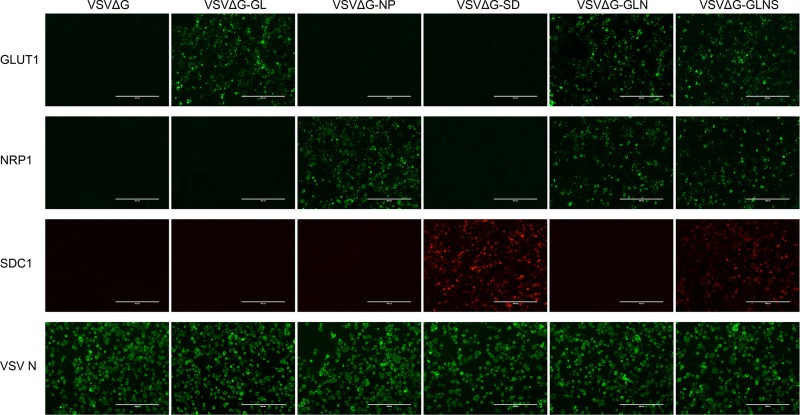
Expression of HTLV-1 receptor molecule(s) on the rVSV-infected target cell surface. The surface expression of GLUT1, NRP1, or SDC1 protein after VSV infection was confirmed by IF. VSV-permissive BHK-21 cells were infected with each G-complemented rVSV (VSVΔG, VSVΔG-GL, VSVΔG-NP, VSVΔG-SD, VSVΔG-GLN, or VSVΔG-GLNS) at an MOI of 0.1. After 3 days of culture, target molecules expressed by the viral genome were stained with FITC-conjugated (GLUT1 and NRP1) or PE-conjugated (SDC1) specific antibodies without any fixation or permeabilization. Intracellular staining of VSV N protein was performed to detect VSV-infected cells, using mouse anti-VSV N MAb followed by FITC-conjugated anti-mouse IgG. The stained cells were observed by fluorescence microscopy and photographed at a constant magnification. Scale bars in panels represent 400 μm.

To evaluate whether rVSVs expressed the HTLV-1 receptor molecule(s) encoded by the viral genome, we examined the protein expression of receptor(s) on the cell surface of infected cells by immunofluorescence (IF) staining. We initially infected BHK-21 cells with G-complemented rVSVs at a multiplicity of infection (MOI) of 0.1, and the cell surface expression of receptor molecules was evaluated by fluorescence microscopy. As shown in Fig. 2, the expression of GLUT1 protein was observed only in the GLUT1-encoding rVSV-infected BHK-21 cells, such as VSVΔG-GL-, VSVΔG-GLN-, and VSVΔG-GLNS-infected cells, but not in VSVΔG-, VSVΔG-NP-, or VSVΔG-SD-infected cells. Similarly, the expression of NRP1 or SDC1 protein was observed only in their encoding virus-infected cells: VSVΔG-NP-, VSVΔG-GLN-, and VSVΔG-GLNS-infected cells expressed NRP1 and VSVΔG-SD- and VSVΔG-GLNS-infected cells expressed SDC1 on their surface, respectively. To confirm G-complemented rVSV infectivity, intracellular immunostaining was used to show comparable expression levels of VSV N protein among rVSV-inoculated cells (VSV N positive, 30 to 40% [Fig. 2, bottom panels]). These results may suggest that receptor molecules were successfully expressed from the encoding viral genome, transported to the cell surface, and incorporated into the progeny virions in rVSV-infected cells. Although the direct detection of incorporated receptor molecule(s) on VSV particles is intriguing, subsequent functional experiments demonstrate that these molecules were incorporated into the VSV particles (see below).

### Specific infection of HTLV-1 Env-expressing cells with rVSVs encoding HTLV-1 primary receptor(s).

**FIG 3:**
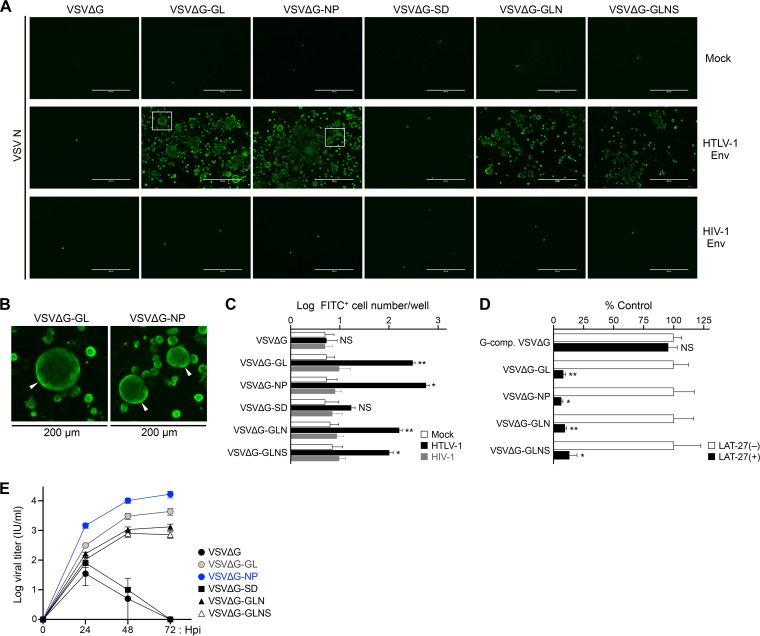
Functional assessment of infectivity and specificity of rVSVs. (A and B) HTLV-1 Env-dependent infectivity of rVSVs was evaluated. BHK-21 cells were initially mock transfected (empty vector) or transfected with expression plasmids for HTLV-1 Env or both HIV-1 Env and Rev. After 24 h, transfected BHK-21 cells were infected with non-G-complemented rVSVs (0.1 ml each). At 4 days of culture, the cells were fixed, and VSV N-specific IF was performed to detect VSV-infected cells. Representative results from each group of rVSV-infected BHK-21 cells are shown in panel A. Areas enclosed with squares are enlarged in panel B. Arrowheads indicate syncytia with enlarged cell size in VSVΔG-GL- or VSVΔG-NP-infected cells. Scale bars in panels A and B represent 400 μm and 200 μm, respectively. (C) Total number of fluorescent cells per well at 24 h post-VSV infection. The data are expressed as the mean ± standard deviation (SD) from three independent experiments. (D) HTLV-1 Env-dependent rVSV infection was assessed by neutralizing assay. BHK-21 cells were initially transfected with expression plasmid for HTLV-1 Env. After 24 h, transfected BHK-21 cells were infected with G-complemented VSVΔG (MOI of 0.1) or non-G-complemented rVSVs, except VSVΔG-SD (0.1 ml each), in the absence or presence of HTLV-1 envelope glycoprotein-specific neutralizing antibody (LAT-27; 10 μg/ml). The cells were stained and examined by VSV N-specific IF at 24 h post-VSV infection. The culture of each virus without LAT-27 was defined as a control. The total number of fluorescent cells per well was determined, and the relative infectivity of each virus was calculated. The data are expressed as a percentage of the control (mean ± SD) from three independent experiments. (E) Viral growth kinetic assay results are shown. HTLV-1 Env-transfected BHK-21 cells were infected with non-G-complemented rVSVs (0.1 ml each). Culture supernatant was collected every 24 hpi until 72 hpi and inoculated into Env-expressing cells again. The cells were analyzed by VSV N-specific IF at 12 hpi, and the total number of fluorescent cells per well was counted to determine the quantitative viral titer (in infectious units per milliliter). The data are expressed as the mean ± SD from two independent experiments. Asterisks in panels C and D represent significant differences versus control (*, *P* < 0.05, **, *P* < 0.01, and NS, no statistical significance, by two-tailed Student's *t* test with equal variance).

To determine the infectivity of all VSV recombinants, we employed HTLV-1 or HIV-1 Env-expressing target cells for rVSV infection. BHK-21 cells were initially mock transfected (empty vector) or transfected with expression plasmids for HTLV-1 Env or both HIV-1 Env and Rev. More than half of HTLV-1 Env-transfected cells expressed the Env protein on the cell surface. Aliquots of these cells were then infected with receptor molecule-bearing rVSVs, including VSVΔG-GL, VSVΔG-NP, VSVΔG-SD, VSVΔG-GLN, or VSVΔG-GLNS, which were not complemented with VSV G protein (non-G-complemented viruses) (28–30). Non-G-complemented VSVΔG was used as an experimental control. At 4 days postinfection (dpi) under culture conditions in the presence of G protein-neutralizing antibodies, the cells were examined by VSV N-specific IF assay to detect and visualize VSV-infected cells. The results showed that non-G-complemented VSVΔG-GL, VSVΔG-NP, VSVΔG-GLN, and VSVΔG-GLNS successfully infected the HTLV-1 Env-expressing cells but did not infect either the HIV-1 Env- or mock (non-Env)-expressing cells (Fig. 3A). In contrast, either VSVΔG- or VSVΔG-SD-inoculated cells showed quite low levels of staining in the IF assay, suggesting these viruses were incapable of infecting HTLV-1 or HIV-1 Env-expressing cells (Fig. 3A). Interestingly, a number of larger cells were observed in rVSV-infected cells (Fig. 3A and B). We previously reported that virus receptor molecules expressed on the surface of rVSV-infected cells mediated cell-cell fusion if the cells also expressed the specific Env protein (i.e., HIV-1 and SIV) (28–30). In accordance, the syncytium formation was induced in rVSV-infected cells expressing both HTLV-1 Env and receptor molecule(s), and we detected larger syncytium-like cells especially in VSVΔG-GL- or VSVΔG-NP-infected cells (Fig. 3A and B).

To evaluate relative infectivity of rVSVs, the number of fluorescent (fluorescein isothiocyanate [FITC]-positive) cells was counted in the transfected BHK-21 cells described above. We detected significant numbers of FITC^+^ cells in HTLV-1 Env-expressing cells infected with VSVΔG-GL, VSVΔG-NP, VSVΔG-GLN, and VSVΔG-GLNS compared to HIV-1 Env- or mock-expressing cells (Fig. 3C). The number of FITC^+^ cells in HTLV-1 Env-expressing cells infected with VSVΔG or VSVΔG-SD was comparable to that in HIV-1 Env- or mock-expressing cells (Fig. 3C). No significant signals were detected in the HIV-1 Env- or mock-expressing cells with any rVSV infection (Fig. 3C). In accordance with Fig. 3A, rVSV infected only HTLV-1 Env-expressing cells inoculated with non-G-complemented viruses, such as VSVΔG-GL, VSVΔG-NP, VSVΔG-GLN, and VSVΔG-GLNS, but not by inoculation with VSVΔG or VSVΔG-SD.

Next, we examined the specificity of rVSV infection by neutralizing assay. As demonstrated above, infectious non-G-complemented rVSVs such as VSVΔG-GL, VSVΔG-NP, VSVΔG-GLN, and VSVΔG-GLNS were pretreated with or without an HTLV-1 Env-specific neutralizing antibody, LAT-27 (33, 34). In addition, G-complemented VSVΔG was used as a control of G-mediated infection at an MOI of 0.1. At 24 h postinfection, the cells were assessed by IF assay. Fluorescence microscopy results revealed that the infectivity of non-G-complemented rVSVs was significantly reduced by treatment with LAT-27 (80 to 90% reduction) compared with mock-treated viruses (Fig. 3D). In contrast, G-complemented VSVΔG infection was barely affected by treatment with LAT-27 (Fig. 3D).

Growth kinetic assays were performed to investigate the replicative abilities of rVSVs in HTLV-1 Env-expressing BHK-21 cells. Non-G-complemented rVSVs such as VSVΔG-GL, VSVΔG-NP, VSVΔG-GLN, and VSVΔG-GLNS underwent replication in the cells during the culture period, whereas non-G-complemented VSVΔG and VSVΔG-SD showed significantly reduced replication (Fig. 3E). Basically, non-G-complemented VSVΔG and VSVΔG-SD did not infect the cells (Fig. 3A and C); however, it is likely that these viruses infected and grew transiently (until 48 h postinfection [hpi]) by residual G complementation, even if the viruses were treated with anti-VSV neutralizing antibodies before inoculation (Fig. 3E).

These results clearly indicated that specific infection was successfully induced through the interaction between HTLV-1 Env protein at the cell surface and receptor molecule(s) incorporated into the VSV particles—particularly the functional association of the Env with GLUT1 or NRP1 proteins. Therefore, VSVΔG-GL and VSVΔG-NP are appropriate as therapeutic candidates for further experiments.

### Elimination of HTLV-1-infected Env-expressing cells by rVSV infection.

**FIG 4:**
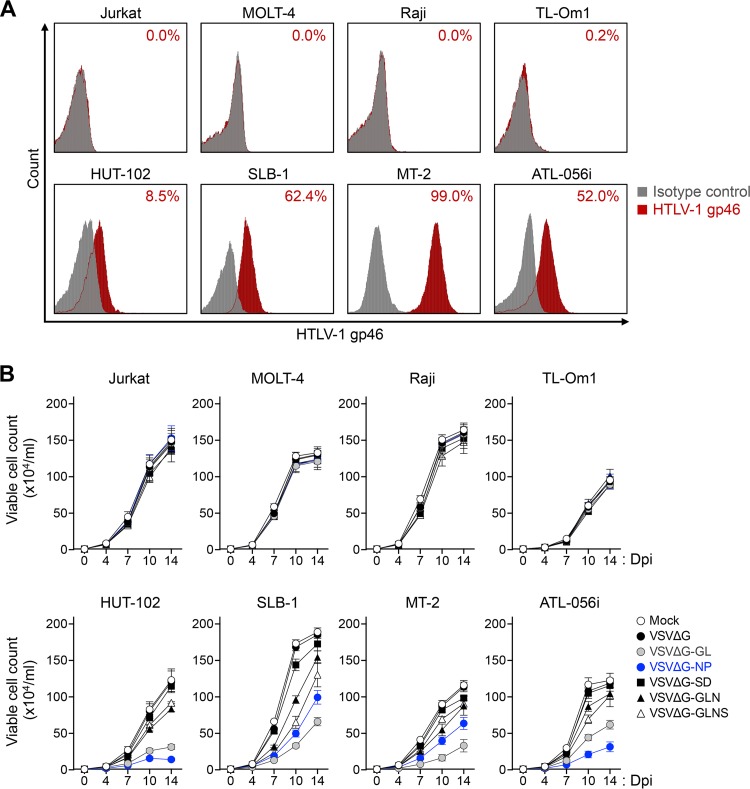
Reduction of HTLV-1-infected Env-expressing target cells by superinfection with rVSVs. (A) The expression level of HTLV-1 Env was analyzed in various HTLV-1-infected or uninfected cell lines. HTLV-1-infected TL-Om1, HUT-102, SLB-1, MT-2, or ATL-056i cells and uninfected Jurkat, MOLT-4, or Raji cells were stained with mouse anti-HTLV-1 gp46 MAb or mouse IgG1 MAb as an isotype control. The stained cells were subjected to flow cytometry. The percentages of HTLV-1 gp46-positive cells are presented in each panel. (B) Results from evaluation of rVSV-mediated killing effects on various HTLV-1-infected or uninfected cell lines are shown. The cell lines described above were infected with G-complemented VSVΔG, VSVΔG-GL, VSVΔG-NP, VSVΔG-SD, VSVΔG-GLN, or VSVΔG-GLNS (MOI of 0.1) or without rVSV (mock) for 14 days. The numbers of viable cells were counted at days 4, 7, 10, and 14 after rVSV infection. Data obtained from triplicate cultures are expressed as the mean ± SD (×10^4^/ml).

We previously demonstrated that rVSVs with viral receptor molecules specifically superinfected and killed target virus-infected cells (28–30). To examine whether similar cytolytic effects could be elicited by infecting rVSVs with HTLV-1 receptor molecule(s), we performed a cell-based killing assay targeting HTLV-1-infected or uninfected cell lines. The infection of rVSV reconstructed here was induced in an HTLV-1 Env-dependent manner. Thus, HTLV-1-infected HUT-102, SLB-1, MT-2, ATL-056i, and TL-Om1 cells or HTLV-1-uninfected Jurkat, MOLT-4, and Raji cells as controls, were analyzed by flow cytometry to confirm their cell surface expression of HTLV-1 Env (Fig. 4A). As expected, HUT-102, SLB-1, MT-2, and ATL-056i cells expressed HTLV-1 Env on their cell surface, while TL-Om1 cells, which are ATL cells that have been reported not to express Env (35), and HTLV-1-uninfected cell lines did not (Fig. 4A).

Next, we infected the cell lines with rVSVs, and the numbers of viable cells were counted every 3 to 4 days for 14 days by trypan blue staining of a portion of replicate cultures. Because G-complemented rVSVs were more infectious (higher titer) than non-G-complemented rVSVs, we used G-complemented rVSVs in this experiment and the following experiments. As shown in Fig. 4B, after superinfection with either VSVΔG-GL or VSVΔG-NP, the numbers of viable cells were sequentially reduced in the HTLV-1-infected cell lines, except for TL-Om1, compared with VSVΔG-superinfected cells in the course of infection (approximately 50 to 80% cell death at 14 dpi). TL-Om1 cells were not susceptible to rVSV infection because this cell line lacks cell surface Env expression (Fig. 4A). Similarly, fewer cytolytic effects were observed in HTLV-1-infected cell lines superinfected with either VSVΔG-GLN or VSVΔG-GLNS (approximately 10 to 30% cell death at 14 dpi). The percentage of killing in VSVΔG-GL-, VSVΔG-NP-, VSVΔG-GLN-, and VSVΔG-GLNS-superinfected, Env-expressing cell lines (HUT-102, SLB-1, MT-2, and ATL-056i cells) was significantly higher (*P* < 0.01 by Student's *t* test) than that in the same cell lines following VSVΔG infection (control).

In contrast, the numbers of viable cells were comparable between the mock-infected and VSVΔG-superinfected cell lines and reached a plateau level at 10 to 14 dpi. No significant effects were observed in the Jurkat, MOLT-4, and Raji cells infected with an rVSV.

These results suggested that HTLV-1-infected Env-expressing cell lines were susceptible to rVSV infection when expressing GLUT1 or NRP1, consistent with the results described in Fig. 3. We also confirmed that VSVΔG-GL and VSVΔG-NP have a greater potential killing activity by superinfection of HTLV-1-infected Env-expressing cells compared with the other rVSVs.

### Therapeutic effects of VSVΔG-NP on HTLV-1 infection in humanized mice.

These findings of high antiviral efficacy against HTLV-1 infection by rVSVs prompted us to perform further studies to determine whether rVSVs inhibit HTLV-1 replication *in vivo*. We previously established an HTLV-1-infected humanized mouse model to study viral pathogenesis and develop antiviral agents (36). Similarly, CD133^+^ human hematopoietic stem cells were used for humanization in this study. Because VSVΔG-NP has a high killing ability against HTLV-1-infected Env-expressing cell lines and a high viral titer *in vitro* compared with VSVΔG-GL (Fig. 3E and 4B), we used VSVΔG-NP to confirm *in vivo* efficacy against HTLV-1 infection. A total of 31 humanized mice reconstituted with CD133^+^ hematopoietic stem cells from five individual donors were used for this study (Table 1).

**TABLE 1:** Characteristics of the humanized mice used in this study

Mouse no.	Sex^*a*^	Donor lot^*b*^	HTLV-1 infection			VSV infection			Endpoint age in wks (+days)^*c*^
			Age in wks (+days)	Inoculated cells	Dose (cells/mouse)	Age in wks (+days)	Inoculated virus	Dose (IU/mouse)	
1^*d*^	M	19138				13 (+0)			17 (+0)
2^*d*^	M	19138				13 (+0)			17 (+0)
3^*d*^	M	19138				13 (+0)			17 (+0)
4^*d*^	F	19138				13 (+0)			17 (+0)
5^*d*^	F	19138				13 (+0)			17 (+0)
6^*d*^	M	19138				13 (+0)	VSVΔG-NP	10 × 10^6^	17 (+0)
7^*d*^	M	19138				13 (+0)	VSVΔG-NP	10 × 10^6^	17 (+0)
8^*d*^	M	19138				13 (+0)	VSVΔG-NP	10 × 10^6^	17 (+0)
9^*d*^	F	19138				13 (+0)	VSVΔG-NP	10 × 10^6^	17 (+0)
10^*d*^	F	19138				13 (+0)	VSVΔG-NP	10 × 10^6^	17 (+0)
11^*e*^	M	24702	17 (+0)	MT-2	2.5 × 10^6^	19 (+3)			25 (+0)
12^*e*^	F	24702	17 (+0)	MT-2	2.5 × 10^6^	19 (+3)			25 (+0)
13^*e*^	M	24717	17 (+0)	MT-2	2.5 × 10^6^	19 (+3)			25 (+0)
14^*e*^	F	24717	17 (+0)	MT-2	2.5 × 10^6^	19 (+3)			25 (+0)
15^*e*^	F	24992	16 (+4)	MT-2	2.5 × 10^6^	19 (+0)			24 (+4)
16^*e*^	M	24702	17 (+0)	MT-2	2.5 × 10^6^	19 (+3)	VSVΔG	10 × 10^6^	25 (+0)
17^*e*^	F	24717	17 (+0)	MT-2	2.5 × 10^6^	19 (+3)	VSVΔG	10 × 10^6^	25 (+0)
18^*e*^	F	24717	17 (+0)	MT-2	2.5 × 10^6^	19 (+3)	VSVΔG	10 × 10^6^	25 (+0)
19^*e*^	M	24992	16 (+4)	MT-2	2.5 × 10^6^	19 (+0)	VSVΔG	10 × 10^6^	24 (+4)
20^*e*^	M	24992	16 (+4)	MT-2	2.5 × 10^6^	19 (+0)	VSVΔG	10 × 10^6^	22 (+3)
21^*e*^	M	24702	17 (+0)	MT-2	2.5 × 10^6^	19 (+3)	VSVΔG-NP	10 × 10^6^	25 (+0)
22^*e*^	M	24717	17 (+0)	MT-2	2.5 × 10^6^	19 (+3)	VSVΔG-NP	10 × 10^6^	25 (+0)
23^*e*^	F	24717	17 (+0)	MT-2	2.5 × 10^6^	19 (+3)	VSVΔG-NP	10 × 10^6^	25 (+0)
24^*e*^	M	24992	16 (+4)	MT-2	2.5 × 10^6^	19 (+0)	VSVΔG-NP	10 × 10^6^	24 (+4)
25^*e*^	F	24992	16 (+4)	MT-2	2.5 × 10^6^	19 (+0)	VSVΔG-NP	10 × 10^6^	24 (+4)
26^*f*^	M	26205	17 (+2)	MT-2	2.5 × 10^6^	19 (+5)			22 (+6)
27^*f*^	M	26205	17 (+2)	MT-2	2.5 × 10^6^	19 (+5)			25 (+0)
28^*f*^	M	26205	17 (+2)	MT-2	2.5 × 10^6^	19 (+5)			26 (+0)
29^*f*^	M	26205	17 (+2)	MT-2	2.5 × 10^6^	19 (+5)	VSVΔG-NP	10 × 10^6^	27 (+1)
30^*f*^	M	26205	17 (+2)	MT-2	2.5 × 10^6^	19 (+5)	VSVΔG-NP	10 × 10^6^	31 (+2)
31^*f*^	M	26205	17 (+2)	MT-2	2.5 × 10^6^	19 (+5)	VSVΔG-NP	10 × 10^6^	33 (+2)

a M, male; F, female.

b Five cord blood donors were used for humanization.

c Age in weeks when indicated mice were sacrificed or died (mice 20, 26, 27, 28, 29, and 30).

d The individual mice correspond to those in Fig. 5.

e The individual mice correspond to those in Fig. 6B to E and 7.

f The individual mice correspond to those in Fig. 6F.

First, we investigated the impact of rVSV infection on blood cellularity *in vivo*. To address this issue, G-complemented VSVΔG-NP or medium (mock control) was inoculated into five intact (HTLV-1-uninfected) humanized mice (Fig. 5A). As shown in Fig. 5B, the numbers of human CD45^+^ lymphocytes, CD19^+^ B cells, and CD4 or CD8 T cells were not affected by G-complemented VSVΔG-NP inoculation compared with the cell numbers in mock controls up to 28 dpi. These results indicate that G-complemented VSVΔG-NP infection does not impinge on the normal hematopoiesis in humanized mice, and they suggest that this rVSV has quite low hematotoxicity *in vivo*.

**FIG 5:**
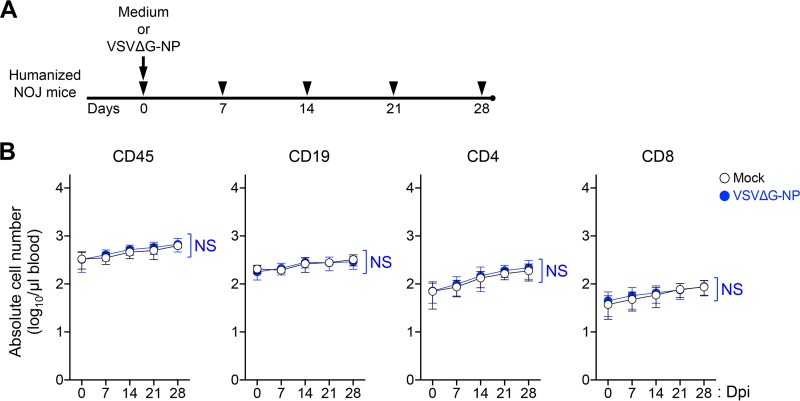
Blood cellularity changes in VSVΔG-NP-inoculated, HTLV-1-uninfected humanized mice. (A) A schematic drawing of the experimental schedule is shown. All NOJ mice were reconstituted with a human immune system by intrahepatic transplantation of human CD133^+^ hematopoietic stem cells into newborn mice. After humanization, mice were administered either medium (mock; *n* = 5) or G-complemented VSVΔG-NP (10 × 10^6^ IU/body; *n* = 5) via a single i.p. injection. Arrowheads indicate the time points for blood collection. (B) Impact of rVSV inoculation on the blood cellularity of humanized mice. The absolute numbers of human lymphocytes are shown. Human CD45^+^ lymphocytes, CD19^+^ B cells, and CD4 or CD8 T cells were routinely analyzed by flow cytometry. The results are presented as the mean ± SD at each time point. Data collected over time from the same mock- or VSVΔG-NP-inoculated mice were compared at matched time points using a two-way ANOVA for repeated measures (NS, no statistical significance).

**FIG 6:**
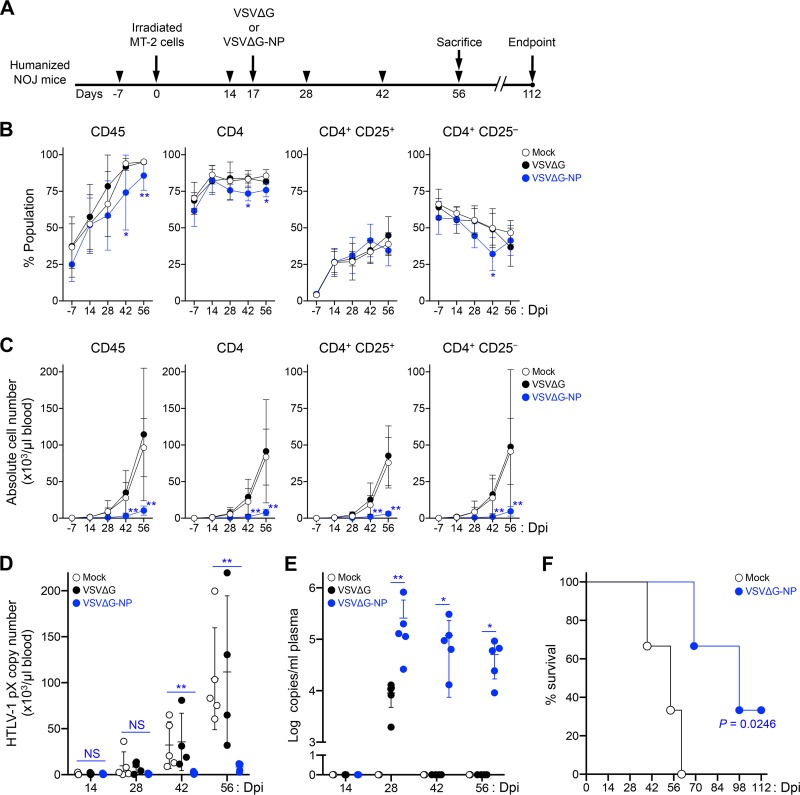
Antiviral effect of VSVΔG-NP on HTLV-1-infected humanized mice. (A) A schematic drawing of the experimental schedule is shown. After humanization, a total of 21 mice were inoculated intraperitoneally with HTLV-1-infected MT-2 cells lethally irradiated (at 0 dpi), and 15 of the mice were used for efficacy evaluation of VSVΔG-NP. HTLV-1-infected mice were administered either medium (mock; *n* = 5) or G-complemented rVSVs (10 × 10^6^ IU/body), including VSVΔG (*n* = 5) and VSVΔG-NP (*n* = 5), by one-shot i.p. injection at 17 dpi. All coinfected mice were observed carefully during the experimental period and sacrificed at 56 dpi. One VSVΔG-inoculated mouse died at 41 dpi. Arrowheads indicate the time points for blood collection. (B and C) Therapeutic effects in the peripheral blood of HTLV-1-infected mice following rVSV inoculation. The frequencies of human lymphocytes are shown in panel B, and the absolute numbers of human lymphocytes are shown in panel C. Human CD45^+^ lymphocytes, total CD4 T cells, and CD25^+^ or CD25^−^ CD4 T cells were routinely analyzed by flow cytometry. CD3^+^ lymphocytes were gated to analyze the populations of CD4 T cells. The results are presented as the mean ± SD at each time point. (D) Quantification of HTLV-1 proviral copy numbers in the peripheral blood of infected mice. HTLV-1 PVL was determined by real-time PCR at 14, 28, 42, and 56 dpi. The HTLV-1 *pX* gene was determined as proviral DNA, and the human HBB gene was used as an internal reference gene. The absolute HTLV-1 proviral copy numbers per microliter of blood were calculated based on both the number of CD45^+^ cells and the PVL at each time point. One dot represents the result from an individual mouse, and the mean ± SD is shown. (E) Quantification of rVSV viral load in the plasma of coinfected mice. Total RNA was isolated from the plasma of each mouse at 14, 28, 42, and 56 dpi. Real-time RT-PCR was carried out to quantify the copy number of VSV-derived viral RNA targeting the L gene. The data are presented as log copies per milliliter of plasma. One dot represents the result from an individual mouse, and the mean ± SD is shown. Undetected samples are all indicated arbitrarily as 10^0^. (F) Kaplan-Meier curve of both mock- and VSVΔG-NP-inoculated mice. For survival analysis, a total of 6 mice were used and inoculated with medium or VSVΔG-NP as described above (mock, *n* = 3; VSVΔG-NP, *n* = 3). All coinfected mice were observed carefully until the experimental endpoint without blood collection. Statistical significance was determined by log rank test, and the resulting *P* value is shown. Asterisks in panels B to D represent significant differences between mock- versus VSVΔG-NP-inoculated mice (*, *P* < 0.05, **, *P* < 0.01, and NS, no statistical significance, by Mann-Whitney *U* test). Asterisks in panel E represent significant differences between VSVΔG-inoculated and VSVΔG-NP-inoculated mice (*, *P* < 0.05, and **, *P* < 0.01, by Mann-Whitney *U* test).

Next, we evaluated the antiviral efficacy of VSVΔG-NP *in vivo*. As shown in Fig. 6A, G-complemented VSVΔG and VSVΔG-NP were each inoculated into five HTLV-1-infected mice at 17 dpi. We also used another five infected mice without rVSV inoculation as mock controls. Peripheral blood mononuclear cells (PBMCs) were routinely collected from all mice on the indicated days, and the cell populations and cell numbers were monitored by flow cytometry. Following rVSV inoculation, the frequencies of human CD45^+^ and CD4 T cells were significantly reduced at 42 and 56 dpi in VSVΔG-NP-inoculated mice compared with mock controls (Fig. 6B). We further analyzed the population of CD4 T cells by dividing them into CD25^+^ or CD25^−^ populations. Interestingly, the frequencies of CD25^+^ in CD4 T cells were comparable between VSVΔG-NP-inoculated and control mice, while the population of CD25^−^ in CD4 T cells was significantly reduced at 42 dpi in the VSVΔG-NP-inoculated mice compared with mock controls (Fig. 6B), suggesting this population may be targeted by the rVSV infection. In addition to the impact of cellularity, the numbers of human CD45^+^, CD4^+^, CD25^+^, or CD25^−^ CD4 T cells were also dramatically decreased in VSVΔG-NP-inoculated mice at 42 and 56 dpi compared with mock controls (Fig. 6C). We also measured HTLV-1 PVL (*pX* copies per 100 cells) in PBMCs routinely collected from each mouse by quantitative PCR. Combined with the results of human cell number analysis, we calculated the absolute proviral DNA copy number per microliter of peripheral blood. As shown in Fig. 6D, the proviral copy numbers in the blood of VSVΔG-NP-inoculated mice were significantly lower at 42 and 56 dpi than in mock controls: the mean proviral copy numbers of VSVΔG-NP-inoculated mice were 1,612 and 8,393, and those of mock controls were 32,162 and 104,413, respectively (Fig. 6D). The results obtained from VSVΔG-inoculated mice were comparable (nonsignificant) to those of mock controls at each time point (Fig. 6B to D).

To evaluate whether VSVΔG-NP propagated *in vivo*, we detected viral RNAs derived from rVSV in the plasma of inoculated mice. We newly established a VSV-specific and sensitive quantitative reverse transcription (RT)-PCR assay targeting the L gene that was not reactive for HTLV-1, to quantify the copy number of viral RNA of VSV (see Table S1 in the supplemental material). As predicted, VSV RNA was successfully detected in the plasma of VSVΔG-NP-inoculated mice at 28, 42, and 56 dpi, but only in VSVΔG-inoculated mice at 28 dpi (Fig. 6E). Similarly, the copy numbers of VSV RNA were higher in the plasma of VSVΔG-NP-inoculated mice over the course of infection (Fig. 6E), suggesting VSVΔG-NP infected the target cells and produced progeny virions in VSVΔG-NP-inoculated mice. VSVΔG propagated transiently by G complementation in coinfected mice, but subsequently VSVΔG did not propagate at all because neither G nor HTLV-1 receptor molecules were encoded in its own genome (Fig. 6E). Furthermore, VSVΔG-NP inoculation also significantly prolonged the survival of HTLV-1-infected mice compared with that of mock controls (Fig. 6F). These findings indicate that VSVΔG-NP exerts a potent therapeutic effect on HTLV-1 *de novo* infection both *in vitro* and *in vivo*.

### Reduced frequencies of HTLV-1 Env-expressing cells in the lymphoid tissues of VSVΔG-NP-inoculated mice.

**FIG 7:**
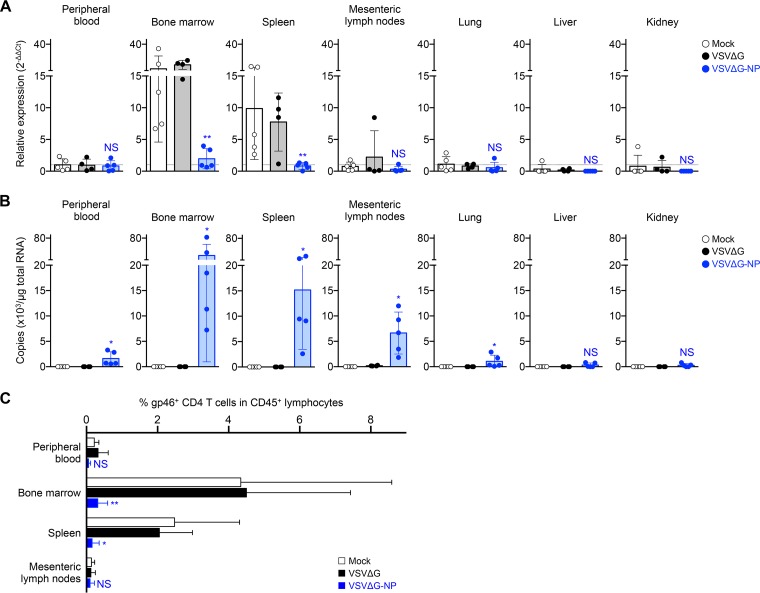
Elimination of HTLV-1 Env-expressing cells in the lymphoid tissues of VSVΔG-NP-inoculated mice. (A) Gene expression profiles of HTLV-1 Env in various organs of coinfected mice. Total RNA was isolated from the peripheral blood, bone marrow, spleen, mesenteric lymph nodes, lung, liver, and kidney of HTLV-1-infected mice following rVSV inoculation at 56 dpi (mock, *n* = 5, VSVΔG, *n* = 4, and VSVΔG-NP, *n* = 5, corresponding with Fig. 6B to E). The expression levels of Env gene were determined by quantitative RT-PCR and were normalized to that of HPRT1. The mean value for the peripheral blood of mock controls is set to 1 as the reference for all specimens. Results are presented as the fold change compared with the reference (dotted line). (B) Quantification of rVSV viral load in various organs of coinfected mice. The total RNA specimens were described above. Real-time RT-PCR was performed to quantify the copy number of VSV-derived viral RNA targeting the L gene. Each dot represents the result from an individual mouse, and the mean ± SD is shown. (C) Cell surface expression of HTLV-1 gp46 was evaluated in the lymphoid tissues of coinfected mice. The bone marrow, spleen, mesenteric lymph nodes, and peripheral blood were harvested from the mice sacrificed at 56 dpi. The frequency of HTLV-1 gp46^+^ CD4 T cells among human CD45^+^ lymphocytes in the tissues was analyzed by flow cytometry. The percentages of HTLV-1 gp46^+^ CD4 T cells in mock-, VSVΔG-, or VSVΔG-NP-inoculated mice are shown. The results are presented as the mean ± SD. Asterisks in panels A and C represent significant differences between mock- versus VSVΔG-NP-inoculated mice (*, *P* < 0.05, **, *P* < 0.01, and NS, no statistical significance, by Mann-Whitney *U* test). Asterisks in panel B represent significant differences between VSVΔG-inoculated and VSVΔG-NP-inoculated mice (*, *P* < 0.05, and NS, no statistical significance, by Mann-Whitney *U* test).

To clarify the effect of VSVΔG-NP on HTLV-1-infected Env-expressing target cells *in vivo*, we first screened the expression levels of HTLV-1 Env in various organs of the coinfected mice described in Fig. 6B to E. As shown in Fig. 7A, relative Env expression in the bone marrow and spleen of mock- and VSVΔG-inoculated mice was relatively high, whereas it was significantly lower in VSVΔG-NP-inoculated mice. In contrast, Env expression levels in the mesenteric lymph nodes, lung, liver, kidney, and peripheral blood of the mice were significantly lower than those in the bone marrow and spleen, and these levels were comparable between the groups lacking statistically significant differences (Fig. 7A).

Next, to investigate whether or not VSVΔG-NP replicated in the various organs of coinfected mice, we have quantified the VSV viral load in the same organs described above. As expected, abundant VSV viral RNA was detected only in the Env-expressing bone marrow and spleen of VSVΔG-NP-inoculated mice; fewer viral RNA copies were detected in the other organs of VSVΔG-NP-inoculated mice (Fig. 7B).

Furthermore, we analyzed the frequencies of HTLV-1 Env-expressing cells in the lymphoid tissues of coinfected mice. Single-cell suspensions were isolated from the bone marrow, spleen, mesenteric lymph node, and peripheral blood of each mouse at 56 dpi and were subjected to flow cytometry. As shown in Fig. 7C, the frequencies of Env-expressing CD4 T cells were markedly reduced in the bone marrow and spleen of VSVΔG-NP-inoculated mice compared with mock controls. The mean frequencies in the bone marrow and spleen of VSVΔG-NP-inoculated mice were decreased by 13.6-fold and 15.5-fold, respectively (Fig. 7C). In contrast, the frequencies of Env-expressing CD4 T cells in the mesenteric lymph nodes and peripheral blood of all mice were similar to basal levels regardless of rVSV inoculation (not statistically significant) (Fig. 7C). Taken together, these results suggest that VSVΔG-NP targeted HTLV-1 Env-expressing cells and exerted its cytotoxicity by rapid viral propagation, which resulted in the elimination of HTLV-1-infected Env-expressing cells in the lymphoid tissues.

## DISCUSSION

To date, a number of recombinant viruses that specifically infect and induce cell death in malignant or virulent cells have been utilized for virotherapy (37–39). These effects were induced by virus-mediated cytopathic mechanisms such as apoptosis, necrosis, and syncytium formation dependent upon fusogenic glycoproteins with target cells (40, 41). It is of benefit to use VSV as a candidate for virotherapy because genetically tractable rVSV vectors with robust cytopathic activity and viral productivity have already been established (42). We previously reported the development of rVSVs that successfully infected and killed HIV-1- or SIV-infected target cells with minimal side effects toward uninfected normal cells (28–30). These recombinant viruses express multiple HIV-1/SIV receptors that are incorporated into viral particles in place of the VSV G protein, restricting the viral tropism to HIV-1/SIV Env-expressing target cells.

Recently, Betancourt et al. reported a CD4-targeting oncolytic rVSV, designated VSV-gp160G, for use against HTLV-1 infection (43). This recombinant virus was retargeted to kill CD4^+^ ATL cells *in vitro* and *in vivo*; however, it did not target the HTLV-1-infected Env-expressing cells on which we focused in this study. Furthermore, the recently developed anti-CCR4 humanized antibody mogamulizumab can rapidly eliminate CCR4^+^ ATL tumor cells (44). Because CCR4 is expressed not only on ATL cells but also on HTLV-1-infected Env-expressing cells, we previously generated a CCL17-fused recombinant toxin targeting CCR4^+^ HTLV-1-infected non-ATL cells (36). However, mogamulizumab also reduced the number of CCR4^+^ regulatory CD4 T cells (45), which was occasionally observed as a severe autoimmune disease (44). Such a reduction in CD4 T cells might also dysregulate autoimmunity in HTLV-1 carriers or HAM/TSP patients (46, 47). To develop a new type of treatment against HTLV-1 infection, the present study demonstrated novel therapeutic rVSV agents based on interactions between viral receptors and Env proteins to specifically target and kill HTLV-1-infected Env-expressing cells.

Previous reports revealed that GLUT1 and NRP1 on the cell surface are involved in HTLV-1 viral entry and membrane fusion via gp46 and gp21 Env glycoproteins (48–50). Here, we demonstrated the HTLV-1 Env-dependent cytolytic activity of rVSVs carrying GLUT1 or NRP1 on its viral particles. Similar to the mechanisms of other VSV recombinants (28–30), infection with G-complemented VSVΔG-GL and VSVΔG-NP moderately reduced the frequency of HTLV-1-infected Env-expressing cells but not uninfected T or B cells under culture conditions (Fig. 4B). Although G-mediated infection occurred in a broad spectrum of cells by G complementation, this side effect is negligible because transient G complementation might allow rVSV to enter cells and kill them only once. In addition to HTLV-1 primary receptor molecules, the HSPGs syndecan and glypican enhanced viral attachment or entry processes (20, 31). Therefore, we investigated the use of the SDC1 gene as an example HSPG gene for viral recombination (Fig. 1). *In vitro* experiments demonstrated both non-G-complemented and G-complemented VSVΔG-SD failed to infect and kill Env-expressing cells, suggesting the expression of SDC1 alone on the viral particle is insufficient for HTLV-1 entry. Taken together and in accordance with previous studies, these findings highlight the need for GLUT1 and NRP1 but not for SDC1 in HTLV-1 entry into or membrane fusion with target cells.

To enhance the infectivity of rVSV against HTLV-1-infected Env-expressing cells, we constructed VSV recombinants with a combination of two or three receptor genes, designated VSVΔG-GLN and VSVΔG-GLNS (Fig. 1). As expected, infection with VSVΔG-GLN or VSVΔG-GLNS induced the expression of target molecules on the infected cell surface (Fig. 2); however, the HTLV-1-specific cytolytic activities of these rVSVs were significantly lower than those of VSVΔG-GL or VSVΔG-NP (Fig. 4B). Unfortunately, viral growth kinetic assays revealed that the titers of VSVΔG-GLN and VSVΔG-GLNS at each time point were lower than those of VSVΔG-GL and VSVΔG-NP (Fig. 3E). Although the mechanisms by which such altered viral activity was observed in VSVΔG-GLN and VSVΔG-GLNS are still unknown, it is likely that receptor molecules expressed by the viral genome physically interacted with each other on the cell surface through the Env protein (51), resulting in the inhibition of viral assembly or release. Further detailed mutagenic analysis will be required to determine the responsible binding sites of each molecule. In addition, because both VSVΔG-GLN and VSVΔG-GLNS encode and express more receptor proteins than the other rVSVs, the extra burden of having additional receptor genes inserted into the genome may have affected the cytolytic abilities of these rVSVs.

In animal experiments, we demonstrated that inoculation of VSVΔG-NP promoted a remarkable therapeutic effect on HTLV-1-infected humanized mice, where the progression of leukocytosis and HTLV-1 proviral copy number in the peripheral blood was significantly inhibited and the survival was significantly prolonged (Fig. 6C, D, and F), indicating this method might be a promising candidate for clinical use with minimal hematotoxicity (Fig. 5). Furthermore, the population of CD25^−^ CD4 T cells was significantly reduced in the peripheral blood of VSVΔG-NP-inoculated mice at 42 dpi (Fig. 6B), suggesting the population was affected by rVSV infection. Interestingly, we previously reported higher transcriptional activation of HTLV-1 provirus in the population of CD25^−^ splenocytes compared with the CD25^+^ population in another humanized mouse model (52), suggesting that CD25^−^ cells are transiently reactivated to express viral genes *in vivo*, which may increase their susceptibility to VSVΔG-NP.

Our rVSVs might function as self-replicating agents because they are replication competent, and therefore their therapeutic potency is also expected to be sustained during infection. We anticipate that rVSV replication is dependent on HTLV-1 replication and that the levels of rVSV replication will decline in parallel with HTLV-1 clearance. Indeed, the copy numbers of VSV RNA in the plasma of VSVΔG-NP-inoculated mice gradually decreased over the course of infection, corresponding to the sequential suppression of HTLV-1 (Fig. 6D and E). These results suggest that VSVΔG-NP infection would decline along with HTLV-1 clearance and may indicate the safe use of VSVΔG-NP in the clinic.

Furthermore, VSVΔG-NP should specifically superinfect and kill target cells that have already been infected with HTLV-1 through virus-mediated membrane fusion following receptor-Env binding. It is also reasonable to assume that such effector mechanisms would target and eliminate HTLV-1-producing (Env-expressing) cells *in vivo*. As expected, we demonstrated a remarkable reduction in the HTLV-1 Env expression levels and the frequency of HTLV-1 gp46-expressing cells in the bone marrow and spleen of VSVΔG-NP-inoculated mice (Fig. 7A and C). Furthermore, we found that the expression of HTLV-1 Env was restricted to cells that belong to the hematopoietic organs of infected mice, such as the bone marrow and the spleen (Fig. 7A and C). In contrast, HTLV-1 gp46-expressing cells were barely detected in the mesenteric lymph nodes and peripheral blood of infected mice, suggesting the expression of HTLV-1 proviral genes was strongly suppressed, similar to observations in HTLV-1-infected patients (53–55). Additionally, the replication of rVSV was observed in all lymphoid tissues of VSVΔG-NP-inoculated mice tested here, and unpredictably, reduced expression of viral RNA was only detected in the mesenteric lymph nodes of replication-incompetent rVSV (VSVΔG)-inoculated mice (Fig. 7B). We believe that such viral RNA is persistent because the genomic RNA of VSV is likely to accumulate at lymph nodes for a long period via phagocytic cells (56) and/or other unknown mechanisms (57). Taken together, these data suggest that VSVΔG-NP targets and kills HTLV-1 Env-expressing cells *in vivo* and that HTLV-1-infected Env-expressing cells derived from hematopoietic organs might be a therapeutic target for this antiretroviral strategy, even if HTLV-1 expression is suppressed in the periphery of HTLV-1-infected individuals (carriers).

It is now recognized that cytolytic viruses can act as immunoactivators (58). Viral internal proteins generated by cytolysis with rVSV can induce cellular immune responses to VSV- and/or HTLV-1-infected cells, which may enhance the elimination of target cells, suggesting the potential use of rVSV for therapeutic vaccines (59–61). We speculate that because the viral membrane of rVSV mostly contains host proteins, they should not induce virus-neutralizing antibodies that inhibit viral spread. Furthermore, existing treatment options can be used to improve or enhance the therapeutic potency of rVSV. For instance, the incorporation of effector enzymes such as thymidine kinase (62), cytosine deaminase (63), and matrix metalloproteinase (64) into viral particles increased cytolytic, motility, and immunostimulatory activities *in vivo*. These techniques should provide an opportunity to develop advanced rVSVs, yielding more suitable virotherapy for clinical use in the future.

In conclusion, we generated a novel rVSV, termed VSVΔG-NP, which targets and suppresses the propagation of HTLV-1 *de novo* infection in our animal model. Our findings also indicate that VSVΔG-NP might be a candidate for unique anti-HTLV-1 virotherapy in HTLV-1 carriers, including as a prophylactic treatment against the development of ATL.

## MATERIALS AND METHODS

### Cells.

BHK-21 cells (Riken BRC Cell Bank, Tsukuba, Japan) were cultured in Dulbecco's modified Eagle's medium (DMEM) (Sigma-Aldrich, St. Louis, MO) supplemented with 10% fetal bovine serum (FBS) and 1× antibiotic-antimycotic (Gibco, Carlsbad, CA). BHK-G cells were cultured in DMEM supplemented with 10% FBS, 750 mg/ml Geneticin, and 0.5 mg/ml tetracycline according to previous studies (30, 32). BHK-G cells were induced to express VSV G under tetracycline-free culture conditions (30, 32). Jurkat and MOLT-4 cells or Raji cells are HTLV-1-negative human T-cell or B-cell lines, respectively. HUT-102, SLB-1, and MT-2 cells are HTLV-1-infected human T-cell lines (65, 66). TL-Om1 and ATL-056i cells are interleukin-2 (IL-2)-dependent immortalized HTLV-1-infected human CD4 T-cell lines established from acute ATL patients (35). These nonadherent cell lines were cultured in RPMI 1640 medium (Sigma-Aldrich) supplemented with 10% FBS and 1× antibiotic-antimycotic, with or without 50 U/ml recombinant human IL-2 (Shionogi Pharmaceutical, Osaka, Japan). All of the cells used in this study were cultured at 37°C in an atmosphere containing 5% CO_2_.

### Mice.

NOD/SCID Jak3-knockout (NOJ) mice were obtained from Kumamoto University (Kumamoto, Japan) and Kyudo Company (Saga, Japan) (67). These mice were maintained under specific-pathogen-free conditions and were handled in accordance with the institutional guidelines for animal experimentation at the National Institute of Infectious Diseases.

### Generation of rVSVs.

To construct rVSV plasmids lacking the G gene and encoding human GLUT1, NRP1, and SDC1, genes encoding these proteins were initially amplified by PCR with the expression vectors for GLUT1 and SDC1 (kindly provided by A. Tanaka, Gunma University) (31) and NRP1 (purchased from OriGene Technologies, Rockville, MD). All rVSV plasmids were generated according to traditional recombination techniques as described previously (27, 30). Briefly, amplified products were excised with restriction enzymes MluI and XhoI (New England BioLabs, Hitchin, United Kingdom) and ligated into G-deleted pVSV-XN2 (a VSV Indiana wild-type vector) digested with the same enzymes using Ligation High version 2 (Toyobo, Osaka, Japan), yielding plasmid vectors of rVSV. The primers containing restriction sites and/or VSV transcription stop-start signal sequences used in this study are listed in Table S2 in the supplemental material.

The rVSVs expressing HTLV-1 receptor molecule(s) complemented with or without G protein were recovered from the plasmid vectors by established methods (27, 30). Briefly, 1 × 10^6^ BHK-21 cells were dispensed into a 10-cm-diameter dish and infected with vTF7-3 at an MOI of 10 to induce T7 RNA polymerase. One hour later, these cells were transfected with the plasmid vectors of rVSV and helper plasmids (pBS-N, pBS-P, pBS-L, and pBS-G) using the *Trans*IT-LT1 reagent (Mirus, Madison, WI) and incubated at 37°C for 48 h. Then, culture supernatants were passed through a 0.2-μm-pore filter to remove the majority of the vaccinia virus and transferred to fresh BHK-G cells previously induced to express G protein. After 48 h, culture supernatants were passed through a 0.1-μm-pore filter to remove residual vaccinia virus completely and then were added to BHK-G cells expressing G protein again. Recovery of infectious viruses was confirmed by microscopy of the VSV-mediated cytopathic effect. Individual plaques were isolated and grown on BHK-G cells previously induced to express G protein. Culture supernatants containing such G-complemented viruses were then harvested and stored at −80°C until use.

To make non-G-complemented virus stocks, G-complemented rVSVs were incubated with 1 × 10^6^ BHK-21 cells seeded onto a 10-cm-diameter dish at an MOI of 10. After adsorption of the viruses, the supernatants were aspirated and the cells were washed with 10 ml FBS-free DMEM per dish at least twice to remove residual input G-complemented viruses. The cells were then incubated in fresh complete DMEM at 37°C for 24 h, and the supernatants were used as stocks for non-G-complemented viruses. The non-G-complemented viruses were incubated with anti-VSV G neutralizing antibodies I1 and I14 (Kerafast, Inc., Boston, MA) at 37°C for 30 min immediately before use of an aliquot of each virus to inactivate the infection mediated by residual G protein.

### Immunofluorescence test.

To assess the surface expression of GLUT1, NRP1, and SDC1 encoded by the VSV genome, 50,000 BHK-21 cells were seeded into each well of a 12-well culture plate, and the following day, the cells were infected with G-complemented rVSVs (MOI of 0.1). At 3 days post-VSV inoculation, the cells were stained with mouse monoclonal antibodies (MAbs) for either GLUT1 or NRP1 (R&D Systems, Minneapolis, MN), followed by incubation with an FITC-conjugated goat anti-mouse antibody (Beckman Coulter, Brea, CA) according to the manufacturer's recommendations. In addition, a phycoerythrin (PE)-conjugated anti-SDC1 MAb (Exbio, Vestec, Czech Republic) was used for the direct staining of cell surface SDC1 protein. The stained cells were observed and photographed with an Evos FL autoimaging system (Thermo Fisher Scientific, Waltham, MA).

To assess the infectivity and specificity of rVSVs, 50,000 BHK-21 cells were seeded into each well of a 12-well culture plate, and the following day, the cells were transfected with a pCAG empty vector or with expression plasmids (29, 68) for HTLV-1 Env or both HIV-1 (SF162) Env and Rev using the *Trans*IT-LT1 reagent according to the manufacturer's protocol. The transfection efficiency was assessed with a green fluorescent protein (GFP) plasmid (pAcGFP1-C1; Clontech, Mountain View, CA); at 24 h after induction, transfection efficiency was >80% in live BHK-21 cells.

After incubation for 24 h, these cells were inoculated with an equal volume (0.1 ml of viral stock) of non-G-complemented rVSVs. Additionally, either G-complemented VSVΔG (MOI of 0.1, as an assay control) or non-G-complemented rVSVs (0.1 ml each), including VSVΔG-GL, VSVΔG-NP, VSVΔG-GLN, and VSVΔG-GLNS, were preincubated with or without the HTLV-1 Env-neutralizing MAb LAT-27 (10 μg/ml) at 37°C for 1 h and then inoculated onto cells transfected with the plasmid for HTLV-1 Env as described above. At 24 h post-VSV inoculation, rVSV-infected cells were determined by the intracellular staining of VSV N protein as described previously (30). The cells were photographed with an Evos FL autoimaging system, and the total number of fluorescent cells in each well was counted by fluorescence microscopy using an Olympus IX70 microscope (Olympus, Tokyo, Japan) to calculate a relative infectivity of rVSV.

### Titration of rVSVs.

Titers of rVSVs were measured by an established method as described previously (30). In brief, for the titration of G-complemented VSVs, viral stocks were diluted serially from 10^−3^ to 10^−5^ with FBS-free DMEM, and then the diluted samples were inoculated onto BHK-21 cells. For the titration of non-G-complemented VSVs, aliquots of viral stocks (0.1 ml each) were inoculated onto BHK-21 cells previously transfected with an expression plasmid for HTLV-1 Env. At 12 h post-VSV inoculation, the cells were fixed and stained, and the total number of fluorescent cells was counted as described above. Titers of each rVSV were calculated and expressed as infectious units (IU) per milliliter. Titers of stocks of G-complemented rVSVs were 2.0 × 10^7^ to 6.2 × 10^7^ IU/ml.

### Cell viability assay.

To evaluate the killing effect of rVSVs on HTLV-1-infected or uninfected cells, 10,000 living cells were seeded into each well of a 12-well plate and then mock infected (medium) or infected with G-complemented rVSVs, including VSVΔG, VSVΔG-GL, VSVΔG-NP, VSVΔG-SD, VSVΔG-GLN, and VSVΔG-GLNS, in triplicate. While the cells were incubated for 14 days, a portion of the culture medium was collected from each well every 3 or 4 days and replaced by an equal volume of fresh medium (28, 36). The cell viability was determined by a standard trypan blue staining method with a Countess automated cell counter (Thermo Fisher Scientific) according to the manufacturer's instructions.

### Establishment of humanized mice with HTLV-1 infection.

Mononuclear cells were isolated from human cord blood (provided by The Japanese Red Cross Kanto-Koshinetsu Cord Blood Bank, Tokyo, Japan) by Ficoll-Histopaque density gradient centrifugation. Human CD133^+^ hematopoietic stem cells were then purified with the CD133 MicroBead kit for hematopoietic tissue (Miltenyi Biotec, Bergisch Gladbach, Germany), according to the manufacturer's instructions. CD133, an early hematopoietic progenitor cell marker, is thought to be ancestral to CD34 in human hematopoiesis (69, 70). Purified cells with a purity of >95% were immediately transplanted intrahepatically into newborn NOJ mice aged 0 to 2 days (1 × 10^5^ cells per mouse). The humanized mice were constructed as described previously; briefly, the humanization process lasted for at least 12 weeks posttransplantation (36). To infect them with HTLV-1, an HTLV-1-producing cell-line, MT-2, was irradiated with 70 Gy by an MX-80Labo X-ray cabinet system (mediXtec Japan, Chiba, Japan). Twenty-one humanized mice were then inoculated with irradiated MT-2 cells intraperitoneally (2.5 × 10^6^ cells per mouse). The absence of residual MT-2 cells in the periphery of all the mice at 14 and 28 dpi was confirmed by PCR with primers specific for a unique integration site of HTLV-1 provirus, as described previously (52). A list of the characteristics of the humanized mice used in this study is provided in Table 1.

### Inoculation of rVSVs into HTLV-1-infected humanized mice.

At 17 days post-HTLV-1 infection, the humanized mice were mock infected (medium) or coinfected with 1.0 × 10^7^ IU per mouse of either G-complemented VSVΔG or VSVΔG-NP by one-shot intraperitoneal (i.p.) injection (5 mice/group in 3 groups). Peripheral blood was routinely collected from each HTLV-1-infected mouse every 14 days and analyzed by flow cytometry and quantitative PCR (see below). All animal experiments were performed in a biosafety laboratory in accordance with the guidelines of the National Institute of Infectious Diseases.

### Flow cytometric analysis.

Peripheral blood mononuclear cells were collected from HTLV-1-infected mice, and following euthanasia, single-cell suspensions of lymphoid tissues were prepared. These cells were prestained with an FcR blocking reagent (Miltenyi Biotec) as described previously (36). For the staining of surface markers, anti-human CD45-phycoerythrin (PE)-Cy7, CD3-FITC, CD3-peridini chlorophyll protein (PerCP)-Cy5.5, CD4-PE, CD8-FITC, CD19-allophycocyanin (APC), and CD25-APC antibodies (Bay Bioscience Co., Ltd., Kobe, Japan) were used according to the manufacturer's protocol. For the staining of HTLV-1 Env, mouse anti-HTLV-1 gp46, mouse IgG1κ (isotype) MAbs, and goat anti-mouse IgG-PE antibody (Abcam, Cambridge, United Kingdom) were used according to the manufacturer's protocol. After staining, flow cytometric analysis was immediately performed on a BD Accuri C6 flow cytometer with CFlow software (Becton Dickinson, Franklin Lakes, NJ), and the collected data were analyzed by FCS Express 4 (De Novo Software, Los Angeles, CA).

### Quantitation of HTLV-1 proviral load.

To determine HTLV-1 infection, the copy numbers of proviral DNA were measured by an established method as described previously (36, 52). In brief, genomic DNA (100 to 200 ng) isolated from suspended cells using the QIAamp DNA blood minikit (Qiagen, Hilden, Germany) was used as a PCR template. Quantitative real-time PCR was performed in duplicate to measure both the copy number of the *pX* region of the HTLV-1 provirus and the human HBB gene as an internal control. The PVL was calculated as [(copy no. of *pX*)/(copy no. of HBB gene/2)] × 100 and expressed as copies per 100 cells. The absolute HTLV-1 proviral copy number per microliter of blood from infected mice was calculated on the basis of the PVL and the absolute number of human CD45^+^ cells (71).

### Development of VSV-specific quantitative real-time RT-PCR.

To develop a novel VSV-specific quantitative real-time RT-PCR assay, we first performed a PCR-based primer/probe screening as described previously (72). In brief, 48 sets of forward and reverse primers specific for the VSV L gene were designed, and 5 sets out of the library were screened by SYBR green-based real-time RT-PCR. 6-Carboxyfluorescein (FAM)-labeled MGB probes were designed for each primer set and then screened by TaqMan-based real-time RT-PCR. The finally identified primer/probe set was as follows: forward, 5′-CTG CCC TTC ATA GGT TTT CG-3′; reverse, 5′-CAT CAA CCT GGT CAA TGC TG-3′; and probe, 5′-FAM-CCA TGG TGG GTT CG-MGBEQ-3′. To quantify the absolute RNA copy number in specimens, synthesized single-stranded RNA (ssRNA) was used as a standard control, and a standard curve from 10^1^ to 10^7^ copies (*R*^2^ > 0.998, percentage of efficiency of >95.0) was generated. Total RNA extraction for fluid or cell specimens was performed using the QIAamp viral RNA minikit or the RNeasy minikit (Qiagen), respectively. All results were corrected by the extraction concentration factor to yield the quantity of viral RNA present in the aliquot of the original specimen.

### Real-time RT-PCR to quantify HTLV-1 Env transcript.

Total RNA extraction for tissue homogenate was performed using an RNeasy minikit according to the manufacturer's instructions (Qiagen). Using the extracted RNA, SYBR green-based one-step RT-PCR was performed as described previously (72). The primers specific for HTLV-1 Env and human hypoxanthine phosphoribosyltransferase 1 (HPRT1 [as an internal control]) are listed in Table S3 in the supplemental material. Relative expression levels were calculated by the conventional threshold cycle (2^−ΔΔ*CT*^) method as described previously (52).

### Statistical analysis.

Statistical analysis was performed using GraphPad Prism 6 (GraphPad Software, La Jolla, CA). The significance of differences was determined by Student *t* tests, Mann-Whitney *U* tests, two-way analysis of variance (ANOVA), and log rank tests. *P* values of <0.05 were considered statistically significant.

### Ethics statement.

All protocols involving human subjects and animal experiments were reviewed and approved by the Institutional Review Board of the National Institute of Infectious Diseases.

### Data availability.

Our findings reported here are available from the corresponding authors upon request.

## Supplementary Material

Supplemental material
