# Supplementary material for: Control of Human T-Cell Leukemia Virus Type 1 (HTLV-1) Infection by Eliminating Envelope Protein-Positive Cells with Recombinant Vesicular Stomatitis Viruses Encoding HTLV-1 Primary Receptor
Source: J Virol. 2018 Jan 30;92(4):e01885-17. doi: 10.1128/JVI.01885-17 (PMC5790936; doi:10.1128/JVI.01885-17)
Supplement: Supplemental material [file supp_92_4_e01885-17__index.html]

Supplemental material 

# Control of Human T-Cell Leukemia Virus Type 1 (HTLV-1) Infection by Eliminating Envelope Protein-Positive Cells with Recombinant Vesicular Stomatitis Viruses Encoding HTLV-1 Primary Receptor

## Supplemental material

- Supplemental file 1 -

  Table S1 (Performance of VSV-specific real-time RT-PCR assay developed in this study.)

  Table S2 (Primers used for rVSV construction.)

  Table S3 (Primers used for real-time RT-PCR assay.)

  XLSX, 46K
